# Perceived Quality of Care for Common Childhood Illnesses: Facility versus Community Based Providers in Uganda

**DOI:** 10.1371/journal.pone.0079943

**Published:** 2013-11-07

**Authors:** Agnes Nanyonjo, Fredrick Makumbi, Patrick Etou, Göran Tomson, Karin Källander, for the inSCALE study group

**Affiliations:** Division of Global Health (IHCAR), Karolinska Institutet, Stockholm, Sweden; 2 Makerere University School of Public Health, Kampala, Uganda; 3 Malaria Consortium, Kampala, Uganda; 4 Medical Management Centre (MMC), Stockholm, Sweden; Tulane University School of Public Health and Tropical Medicine, United States of America

## Abstract

**Objective:**

To compare caretakers’ perceived quality of care (PQC) for under-fives treated for malaria, pneumonia and diarrhoea by community health workers (CHWs) and primary health facility workers (PHFWs).

**Methods:**

Caretaker rated PQC for children aged (2-59) months treated by either CHWs or PHFWs for a bought of malaria, pneumonia or diarrhoea was cross-sectionally compared in quality domains of accessibility, continuity, comprehensiveness, integration, clinical interaction, interpersonal treatment and trust. Child samples were randomly drawn from CHW (419) and clinic (399) records from eight Midwestern Uganda districts. An overall PQC score was predicted through factor analysis. PQC scores were compared for CHWs and PHFWs using Wilcoxon rank-sum test. Multinomial logistic regression models were used to specify the association between categorized PQC and service providers for each quality domain. Finally, overall PQC was dichotomized into “high” and “low” based on median score and relative risks (RR) for PQC-service provider association were modeled in a “modified” Poisson regression model.

**Results:**

Mean (SD) overall PQC was significantly higher for CHWs 0.58 (0 .66) compared to PHFWs -0.58 (0.94), p<0.0001. In “modified” Poisson regression, the proportion of caretakers reporting high PQC was higher for CHWS compared to PHFWs, RR=3.1, 95%CI(2.5-3.8). In multinomial models PQC was significantly higher for CHWs compared to PHFWs in all domains except for continuity.

**Conclusion:**

PQC was significantly higher for CHWs compared to PHFWs in this resource constrained setting. CHWs should be tapped human resources for universal health coverage while scaling up basic child intervention as PQC might improve intervention utilization.

## Introduction

In order to address childhood mortality, scarcity of human resources for health and inequalities in access to health care, approaches like the integrated community case management of childhood illnesses (iCCM) have been recommended by the World Health Organisation [1,2,3]. iCCM entails use of community health workers (CHWs) in the management of uncomplicated childhood illnesses including malaria, pneumonia, diarrhoea and newborn referral [3,4,5]. Despite the ability of community based interventions to improve access to life saving interventions, improvement is still needed in the quality of health care delivered to sick children, highlighting the dire need to strengthen health systems [6,7,8]. Many health initiatives today are faced with questionable ability to be scaled up with quality [9], making quality an important aspect of programme evaluation.

Literature on the quality of health care concept has previously emphasised the association between health infrastructure and quality received but more attention is needed in applying this concept to individual users [10]. This is crucial because the effectiveness of quality of health care is a factor of both clinical and interpersonal care effectiveness. Factors not counting as infrastructure, such as poor performance of health workers, affect quality received [11]. Total quality assessment requires structure, process and outcome measure evaluation [10,12,13,14]. Structure refers to the availability and well organisation of resources (infrastructure, material and human) acting as channels through which care is delivered. Process reflects the actual process undergone during a clinical interaction encounter. Outcomes are the changes in the receiver’s health status or any desired outcomes occurring as a consequence of health provider-receiver interaction [10,12]. It is not possible to bridge health inequities by merely improving access [15,16]. User perception of quality of health care is known to drive utilization of health interventions [17,18,19]. In the context of user satisfaction, technical improvements and continuity of care alone are known to improve the ratings of perceived quality of care (PQC) [18,20,21]. 

In Uganda, basic primary health care for children is obtained from CHWs and primary health facility workers (PHFWs). Previously the quality of primary health care for children has been evaluated in both integrated management of childhood illnesses provided at health facilities and iCCM provided in the communities [22,23,24,25,26]. This evaluation was however limited in that it was hinged on structural and provider assessments and not PQC at the user level. The PQC received from CHWs has not been established previously. Evaluation of PQC is important since it affects caregiver demand and utilization of CHW services, which subsequently could improve health outcomes in sick children through more prompt care for uncomplicated cases and increased care quality for more complicated cases at health facilities through decreased staff workload [27]. Furthermore the private sector has been the largest provider of health care for sick children in Uganda followed by the formal sector [28]. In order for iCCM to be embraced by the communities as an alternative source of care, its PQC must be comparable to that of the standard care in the formal sector. This study reports on a cross-sectional comparison of the PQC among caretakers of children treated for malaria, pneumonia and diarrhoea by CHWs and by primary health facility workers (PFHWs). 

## Methods

### Ethics statement

Ethical approval for the study was obtained from the Institutional Review Board of Makerere University School of Public Health and the Uganda National Council of Science and Technology (HS 958). Written consent was obtained from all caretakers before conducting the interviews.

### Study design

A comparative cross-sectional survey of caretakers of children visiting CHWs and PHFWs for management of malaria, pneumonia and diarrhea.

### Study settings and context

Between August and September 2011, a survey was undertaken in eight Midwestern Uganda districts. The districts were estimated to have a population of 2.2 million people of which 18% were children under five. Health seeking behaviour of the people in the study areas ranges from treatments at home to formal and informal health providers outside the home, including drug shops, clinics, health facilities and traditional healers. In one study it was documented that 62.5% of children seeking care outside the home were first managed by a drug shop or private clinic [28].

**Figure 1:**
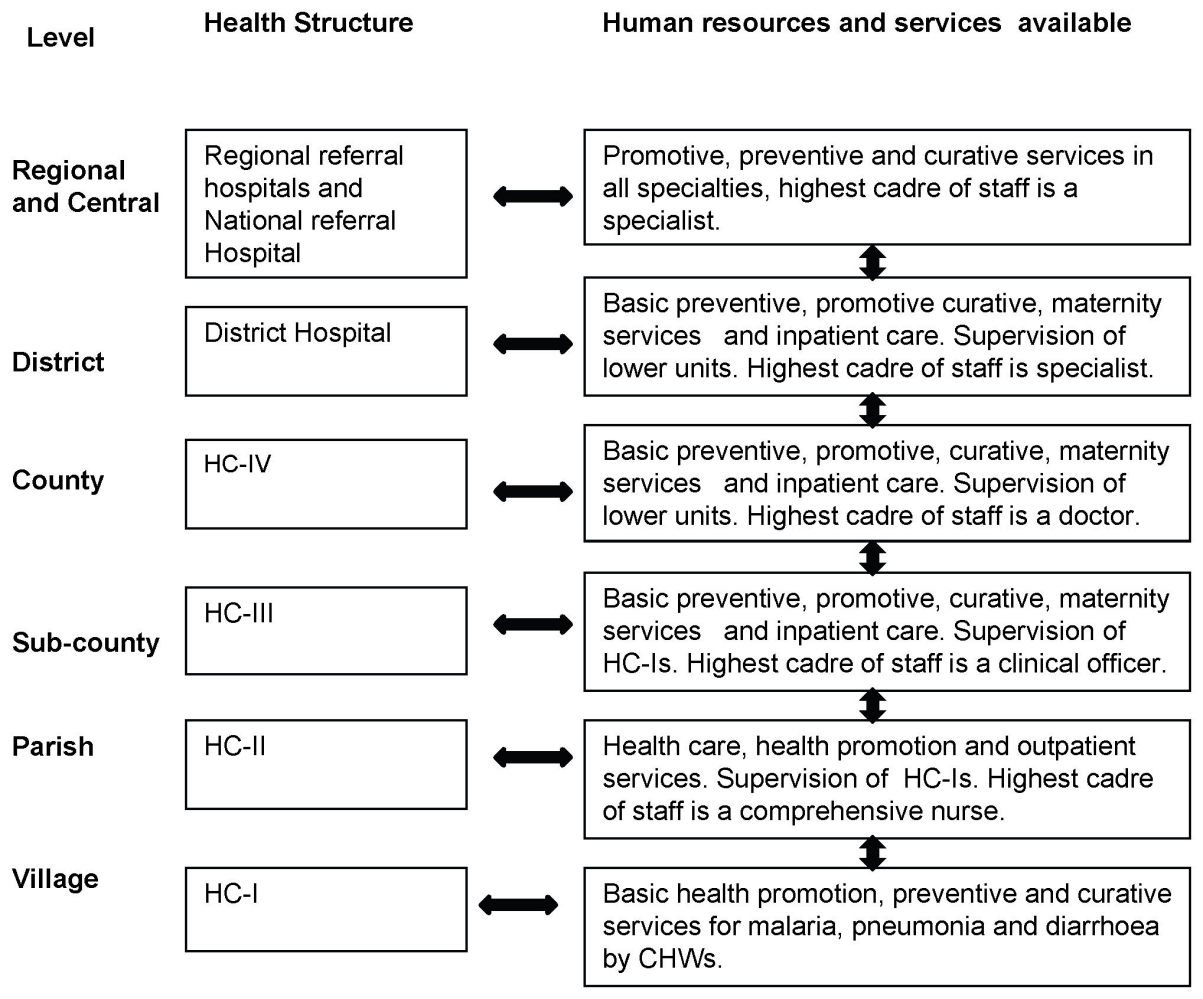
A diagrammatic depiction of the health system hierarchy in Uganda.

 During the study period approximately 6674 CHWs trained on iCCM were operating in an area which was served by 192 government owned, 51 private not for profit and 33 private health centres (HCs). In Uganda’s health system hierarchy (Figure 1), the lowest HC operating at the village level (HC-I) is the CHW who works from home [29]. The first HC with a physical structure is at the parish level (HC-II), followed by HC III at the sub-county level and HC-IV at the county level. Hospitals operate at district level. Majority of the HCs with a physical structure in the study area were level II and III. The HCs from level II-IV are herein referred to as primary health facilities. 

Under Uganda’s village health team (VHT) strategy, 5-6 CHWs are trained to carry out health promotion and education activities, including follow-ups for pregnant women and sick newborn referrals. Two CHWs in each village are additionally trained on iCCM. A typical CHW is trained on iCCM for six days, oversees 20-30 households and is responsible for diagnosing and treating children suffering from uncomplicated malaria, pneumonia and diarrhoea, as well as referral of newborns with danger signs. CHWs are provided with colour coded Artemether/Lumefantrine combination to treat children aged 4 -59 months confirmed to have uncomplicated malaria through a rapid diagnostic test ; amoxicillin to treat uncomplicated pneumonia among children aged 2 -59 months and zinc and oral rehydration salts to treat uncomplicated diarrhoea in children aged 2 -59 months. Children showing danger signs are referred to the nearest health facility. Pre-referral rectal Artesunate is administered to children with danger signs attributable to severe malaria and pre-referral amoxicillin is provided to children with signs of severe pneumonia. The CHWs are supervised by staff from the nearest primary health facility. The PHFWs receive refresher courses on IMCI as part of their orientation to train and supervise the CHWs.

HC-II’s and HC-III’s mainly constitute the primary health facilities in Uganda. A HC-III provides basic preventive, promotive and curative care and provides support supervision for the community and HC-IIs under its jurisdiction with a team consisting of a clinical officer and nurses. With respect to malaria, pneumonia and diarrhoea among under-fives, basic laboratory services are available for diagnosis of the three diseases. HC-IIIs are recommended to treat uncomplicated malaria with Artemether/Lumefantrine combination, uncomplicated pneumonia with vitamin A and either amoxicillin or a combination of cotrimoxazole and procaine penicillin forte if necessary. Watery diarrhoea is treated with ORS and zinc at this level. The HC-IIs provide the first level of interaction between the formal health sector and the communities. HC-IIs only provide outpatient care through an enrolled comprehensive nurse who also acts as a supervisor for CHWs. HC-IIs are required to treat uncomplicated malaria with Artemether/Lumefantrine combination, uncomplicated pneumonia with vitamin A and amoxicillin and watery diarrhoea with ORS and zinc. 

Study participants were caretakers of children aged 4-59 months who had received treatment for malaria from a CHW or PHFW and children aged 2-59 months who had received treatment for pneumonia or diarrhoea from either CHWs or PHFWs in sub-counties served by both CHWs and PHFWs. Caretakers were mainly parents and grandparents of the sick children.

### Sample size estimation

Assuming an average PQC of 2.7 and standard deviation 0.82 for PHFWs (satisfaction with quality of health care for patients attending the outpatient clinic in Mulago hospital Uganda from Nabbuye-Sekandi et al [21]); a sample size of 158 participants were required in order to detect a 30% difference in mean PQC for CHWs and PHFWs with 90% power at 5% significance. In order to adjust for a cluster effect of 2 and a loss to follow up of 10%, a sample of 348 participants was required in each group.

### Sampling method

Using computerised random sampling 30% of the 72 sub-counties with CHWs trained on iCCM were sampled (for logistical purposes). Using cluster sampling with probability proportionate to cluster size, 98 villages were selected from the sub-counties. All CHWs and their supporting primary health facility units for the selected villages were included in the study. Among the children treated by each selected CHW within 2 weeks of interview, a maximum of 3 were sampled by a trained research assistant through systematic random sampling of the register. Samples from the primary health facility units were drawn through systematic sampling from a list compiled from the outpatient register including only children with uncomplicated malaria, pneumonia and or diarrhoea seen in the past two weeks. The children sampled from the registers were restricted to ages 2-59 months for diarrhoea and pneumonia and 4-59 months for malaria. Equal numbers of children were sampled from the CHWs and PHFWs working in the primary health facility units of the same catchment area. Prior to data collection, out-patient department staff were asked to record contact information for caretakers of all under fives who did not mind having a follow up interview at home. The caretakers of children sampled from the records were then traced and interviewed.

### Study instruments

Drawing from Donabedian’s criteria for quality assessment [12] a last visit based quality of healthcare assessment tool (available from first author on request) was used. The tool was adapted from the primary care assessment survey (available on request at http://icrhps.tuftsmedicalcenter.org/research/thi/pcas.asp) and the Uganda service provision assessment survey [30,31] and was administered to the caretakers by trained research assistants. Primary care assessment survey and a similar tool known as the primary care assessment tool have previously been tested for validity and reliability [30,32,33,34]. The tool was translated into the native languages before pilot testing. The tool measured quality domains (table 1) using weighted scale items assumed to have equal relationship to the quality concept (summary scales) being measured. Each summary scale had a set of items (2-8) that prompted participants to rate their experiences with quality of health care. The rating scales varied from a minimum of 0-2 to a maximum of 0-10.

**Table 1:** Domains, summary scales and scale items used in measurement of perceived quality of care on the last visit to the health worker.

Domain Area	Definition	Domain summary scale	Scale item/content
Structure of the health care	Health system organisational factors that act as conduit through which care is offered	Financial access	Measure of the amount of money spent on treatment including user fees during an illness episode
		Organizational access	Availability of staff and services and convenience of location of the health structure
		Longitudinal continuity	Duration of contact between the health provider and user
		Visit based continuity	Ongoing care for the same episode of illness
Process	Processes refers to what is done to the user during a health care delivery and receipt relationship	patient competence (comprehensiveness)	Provider knowledge of the patient
		Preventive Counselling (comprehensiveness)	Discussion of preventive measures with the user
		Integration of health services	Providers role in coordinating referral and receiving feedback in from referred patients/caretakers
		communication	Ability to probe for symptoms give feedback and assist in making treatment decisions
		Physical exam	Thoroughness of the physical examination
		Interpersonal treatment	Patience, friendliness , respect and giving quality time to patient
		Trust	Integrity , patience and role of health provider as patient’s agent
Outcome	Outcomes are occurrences arising as a result of health care received and are aimed at maximizing benefits according to the user’s needs	Measures of outcome	
		Satisfaction	
		Health status	Change in health status and quality of life attributable to health service provided
		Enablement felt	Empowerment to have behavioural change.

### Statistical analysis

The overall objective of the analysis was to compare PQC scores for caretakers of children who received treatment from CHWs to those received treatment from PHFWs. Using the primary care assessment survey scoring guidelines (available on request at http://icrhps.tuftsmedicalcenter.org/research/thi/pcas.asp) reverse coding was done for scale items with questions asked in a negative way to achieve consistency in directionality. Items with higher rating score values were recalibrated to ensure equal weights for all items contributing to the same summary scale. Recalibration ensured that equal total scale score variance contribution assumptions underlying valid linear summated rating scales are fulfilled. Raw scale scores were calculated for each scale by summing across scale items for participants responding to over 50% summary scale items. In case of missing values and a response rate above 50% scale items, the median was used to impute data before raw scale score calculation. Raw scale scores were converted into transformed scale scores on a scale of 0-100 using the formula recommended by the primary care assessment survey scoring guidelines.

Transforemed.scale.score=(mean.item.value−lowest.possible.value)÷possible.item.value.range×100

Domain summary scale scores with adequate sample size in both groups (9/11) were used to predict an overall PQC score through factor analysis. The two excluded items included integration and longitudinal continuity. Chi-square tests were used to a) compare socio-demographic characteristics and categorical summary scale items between the two groups and b) compare categorical treatment outcomes between the two groups. PQC for each summary scale, the overall predicted quality scores, and stated overall satisfaction were compared between the two groups using Wilcoxon rank-sum test as data were highly skewed. The lower, median (middle two) and upper quartiles of each summary scale score were categorised as low, medium and high-quality, respectively. Multinomial logistic regression for categorical outcomes was used to assess the association between PQC for each summary scale and service providers while controlling for potential confounders. Robust standard errors with adjustment for clustering at the village and primary health facility levels were used to account for possible correlation between caretakers of children visiting the same health worker. Given the fact that independent multinomial models yielded high relative risks and consistent directions of association between PQC and service providers among the three quality categories, the overall predicted quality scores were dichotomised into “high quality” and “low quality” using a median split. A modified Poisson regression model was then used to adjust for these high relative risk ratios and establish the association between the overall predicted PQC and service providers. The modified Poisson regression model with a sandwich error term can be used to estimate relative risks with efficiency and consistency. The model uses a logarithm link and hence the estimates are robust to omitted covariates [35,36,37]. Questions on ownership of household items, household construction materials, sanitation infrastructure and means of transportation were used to create a single indicator representing household wealth through principal components analysis. The wealth indicator of individual households ranked from lowest to highest was divided into the upper, middle and lower terciles which represent the socioeconomic status. The individual indicators that weighted heaviest in the analysis were house construction materials (roof, walls and floor material) and type of toilet facility used. All analysis was done in STATA version 12.

## Results

Data were drawn from caretakers of children with complete data and whose last visit was with a PHFWs (376/399; 94.2%) or CHWs (377/419; 90.0%). Of the complete observations, 3.0% had at least one summary scale item value imputed and exclusion of these results would not significantly affect the sample size. All study participants were recruited from 98 villages in 24 sub-counties. The group of caretakers who last visited CHWs had significantly more missing overall predicted quality scores (10.0%) relative to the group that last visited PHFWs (5.8%, p=0.02). Majority (82%) of caretakers visiting CHWs had historical contact with the PHFWs in the last month, while only 54.5% of caretakers visiting PHFWs had been to a CHW with a sick child (p<0.001).

Overall there were no observed differences in age and sex between caretakers visiting CHWs or PHFWs (table 2). Caretakers visiting the CHWs had a significantly higher socioeconomic status compared to those visiting the PHFWs .The caretakers of children managed by PHFWs were also more likely to have education levels beyond primary education. The proportion of children with diarrhoea was significantly higher in children visiting PHFWs, while no significant differences were observed for malaria and pneumonia. They also often had overall predicted quality scores falling in the high or medium category compared to low (table 2). With respect to PHFWs, higher proportions of overall predicted quality scores fell in the medium category for HC-IIs and HC-IIIs (60.7% and 45%) respectively, while more fell in the low category for HC-IVs (79.5%; p<0.001).

**Table 2:** Distribution of basic characteristics and overall perceived quality scores between caretakers of children treated by PHFWs and CHWs.

Characteristic	PHFWs Number (%)	CHWs Number (%)	Total %	p-value
Sex				0.402
Female	359 (90.9)	383 (92.5)	742 (91.7)	
Male	36 (9.1)	31 (7.5)	67 (8.3)	
total	395	414	809	
Age				0.792
0-19	38 (9.7)	33 (8.0)	71 (8.8)	
20-29	306 (78.5)	328 (79.4)	634 (79.0)	
40+	46 (1.8)	52 (12.6)	88 (12.2)	
total	390	413	803	
education level				0.032*
None	69 (17.3)	61(14.6)	130 (15.9)	
Primary	240 (60.1)	288 (68.7)	528 (64.5)	
Secondary and beyond	90 (22.6)	70 (16.7)	160 (19.6)	
Total	399	419	818	
social economic status				0.020*
Lower (1/3)	150 (37.6)	123 (29.4)	273 (33.4)	
Middle (1/3)	132 (33.1)	141 (33.6)	273 (33.4)	
Upper (1/3)	117 (29.3)	155 (37.0)	272 (33.2)	
Total	399	419	818	
malaria diagnosis				0.073
No	104 (26.1)	85 (20.7)	189 (23.4)	
Yes	295 (73.9)	325 (79.3)	620 (76.6)	
Total	399	410	809	
pneumonia				0.089
No	280 (70.2)	311 (75.5)	591 (72.9)	
Yes	119 (29.8)	101 (25.5)	220 (27.1)	
total	399	412	811	
diarrhoea				0.013*
No	299 (75.0)	344 (82.1)	643 (78.6)	
Yes	100 (25.0)	67 (17.9)	175 (21.4)	
total	399	419	818	
Quality category				<0.001*
low	171 (45.5)	18 (4.8)	189 (25.1)	
medium	179 (47.6)	196 (52.0)	375 (49.8)	
high	26 (6.9)	163 (43.2)	189 (25.1)	
Total	376	377	753	

* Significant p-value

Summary scales for organisational and financial access varied greatly between PHFWs and CHWs due to differences in constituent items summarised in table 3. Organisational access was generally perceived to be higher among caretakers of children treated by CHWs compared to those treated by PHFWs as explained by shorter travel distances, waiting times and better access hours. Financial access scores were lower for the PHFWs with higher proportions of caretakers contemplating cancelling treatment visits and skipping medications due to expenses involved.

**Table 3:** Association between access measurements and service providers.

Characteristic	PHFWs Number (%)	CHWs Number (%)	Total (%)	P-Value
Organizational access				
Travel time				
Less than 30 min	151 (37.9)	328 (78.3)	479 (58.6)	<0.001*
0.5-1 hours	117 (29.4)	71 (16.9)	188( 23.0)	
1 - 2 hours	86 (21.6)	17 (4.1)	103(12.6)	
More than 2 hours	44 (11.1)	3 (0.7)	47 (5.7)	
Total	398	419	817	
More opening hours needed				
Early in the morning No	227 (61.8)	353 (84.2)	580 (73.8)	<0.001*
Yes	140 (38.1)	66 (15.7)	206 (26.2)	
Total	367	419	786	
In the evenings No	313 (91.2)	390 (93.1)	703 (92.4)	0.420
Yes	29 (8.4)	29 (6.9)	59 (7.6)	
Total	342	419	761	
On weekends No	270 (76.3)	413 (98.6)	683(88.4)	<0.001*
Yes	84 (23.7)	6 (1.4)	90 (11.6)	
Total	354	419	773	
None No	236 (66.7)	105 (25.1)		<0.001*
Yes	118 (33.3)	313 (74.9)		
Total	354	418	772	
Waiting time				
≤ 5 min	142 (35.7)	396 (94.5)	538 (65.8)	< 0.001*
6 -30 min	107 (26.9)	20 (4.8)	127 (15.5)	
31 -60 min	21 ( 5.3)	1 (0.2)	22 (2.7)	
> 60 min	128 (32.2)	2 (0.5)	130 (15.9)	
Total	398	419	817	
Financial access				
Visit costs expensive No	358 (89.7)	414 (98.8)	772 (94.4)	<0.001*
Yes	41(10.3)	5 (1.2)	46 (5.6)	
Total	399	419	818	
Drugs skipped due to cost No	41 (10.3)	13 (3.1)	54 (6.6)	<0.001*
Yes	356 (89.7)	406 (96.9)	762 (93.4)	
Total	397	419	816	

* Significant p-value

There were differences in PQC scores for the two groups, both for the overall predicted score and for individual summary scale items or domains (table 4). Overall, CHWs’ ratings were significantly higher than PHFWs ratings in the areas of clinical interaction, patient competence, preventive counselling, examination, communication and interpersonal treatment. Although children treated by CHWs were more likely to see the same person on different visits for the same illness episode the continuity of care was rated low. Additionally children treated by PHFWs were more likely to have had the same provider for longer durations compared to those treated by CHWs. The proportion of children referred to seek treatment from a higher level facility was significantly higher for CHWs (14.3%) compared to the PHFWs (2.0%, p<0.0001) but no significant differences existed in perceived integration of health services. 

**Table 4:** Difference in perceived quality of care between caretakers of children visiting community health workers and primary health facilities.

DOMAIN ITEM	PHFWs		CHWs		
Structure	Mean (SD)	Median (IQR)	Mean (SD)	Median (IQR)	Ranksum p-value
Organizational access	52.4 (21.8)	53.3 (40.0- 66.7)	81.2 (14.8)	80.0 (73.3-3.3)	<0.0001*
Visit based continuity	38.3 (21.3)	40 (20.0-40.0)	17.2(17.8)	20.0 (0.0-20.0)	<0.0001*
Longitudinal continuity	64.6 (35.6)	75.0 (25.0-100)	42.0 (26.4)	50.0 (25.0-50.0)	<0.0001*
Financial access	66.5 (23.9)	60.0 (50.0-80.0)	85.0 (17.9)	90.0 (80.0-100)	<0.0001*
Process-comprehensiveness					
Patient competence	52.4 (22.2)	53.7 (33.7-68.7)	70.3 (17.2)	72.5 (58.7-83.7)	<0.0001*
preventive counselling	51.8(38.4)	60.8 (21.6-100)	76.5 (30.4)	80.4 (60.8-100)	<0.0001*
Process-clinical interaction					
physical examination	61.9 (23.9)	60.0 (60.0-80.0)	80.8 (16.2)	80.0 (80.0-100)	<0.0001*
Communication	60.7 (18.1)	63.3 (53.3-70.0)	76.7 (13.0)	76.7 (66.7-86.7)	<0.0001*
Interpersonal treatment	61.3 (21.2)	60.0 (48.0-80.0)	80.7 (15.2)	80.0 (68.0-96.0)	<0.0001*
Trust	69.6 (17.1)	67.8 (57.1-82.1)	79.4 (13.2)	78.6 (71.4-89.3)	<0.0001*
Process- integration					
Integration	57.7 (23.4)	58.3 (46.7-73.3)	57.6 (22.4)	53.3 (40.0-73.3)	0.2820
overall quality score	-0.58 ( 0.94)	-0.50 ( -3.77- 1.82)	0.58 (0 .66)	0.65 ( -1.66- 1.79)	<0.0001*
Satisfaction (outcome)	24.4 (16.9)	16.7 (16.7-33.3)	14.5 (11.4)	16.7 (0.0-16.7)	<0.0001*

* Significant p-value

With regards to visit outcomes, mean stated satisfaction with the entire visit was higher for PHFWs visits compared to CHWs visits (table 4). Caretakers of children treated by CHWs registered higher cure prevalence and behavioural change as a result of advice provided on the last visit, compared to caretakers visiting PHFWs (table 5). 

**Table 5:** Distribution of visit outcomes between CHWs and PHFWs.

outcome	PHFWs	CHWs	Total	P-value
	Number (%)	Number (%)	Number (%)	
Child recovered				<0.001*
Yes	254 (71.7)	346 (85.2)	600 (78.9)	
No	100 (28.2)	60 (14.8)	160 (21.0)	
Total	354	406	760	
Required extra treatment				0.362
Yes	25 (7.2)	32 (9.1)	57 (8.1)	
No	322 (92.8)	320 (90.9)	642 (91.8)	
Total	347	352	699	
Child died	0 (0)	0 (0)	0	N/A
Had complications				0.302
Yes	6 (1.7)	3 (0.9)	9 (1.3)	
No	338 (98.3)	347 (99.1)	685 (98.7)	
Total	344	350	694	
Still on treatment				0.184
Yes	30 (7.5)	22 (5.2)	52 (6.4)	
No	369 (92.5)	397 (94.7)	766 (93.6)	
Total	399	419	818	
Behavioural change following treatment				
Sleeping under a net				
Yes	265 (66.7)	330 (79.3)	595 (73.2)	<0.001*
No	132 (33.2)	86 (20.6)	218 (26.8)	
Total	397	416	813	
Drinking clean water				
Yes	242 (60.8)	310 (74.7)	552 (67.9)	<0.001*
No	156 (39.2)	105 (25.3)	261 (32.1)	
Total	398	415	813	
Feeding the sick child				
Yes	266 (67.0)	330 (79.1)	596 (73.2)	<0.001*
No	131 (33.0)	87 (20.9)	218 ( 26.8)	
Total	397	417	814	

* Significant p-value

In multinomial regression models for the separate quality domains where potential confounders were adjusted for (table 6), the model for organizational access had the highest relative risk ratios for quality compared to the models for other summary scales (domains). In the “modified” Poisson regression model (table 7), adjusting for potential confounding factors with overall PQC categorised as “high” and “low”,the proportion of caretakers reporting high PQC was higher for CHWS compared to PHFWs, RR=3.1 (95%CI(2.5-3.8).

**Table 6:** Multinomial logistic regression model of association between categorised perceived quality of care and service provider for the overall perceived quality score and for each domain (N= 753).

Domain/Scale item	Quality category	CHWs versus PHFWs (unadjusted)	CHWs vs PHFWs (unadjusted)
Financial access	Low quality	1.0	1.0
	Medium quality	4.6 (3.17-6.85)	5.2 (3.5-8.01)
	High quality	7.3 (4.94-10.88)	7.8(5.27-11.01)
Organizational access	Medium versus low	17.1(10.17-28.93)	19.2 (10.88-33.80)
	High versus low	78.5 (38.97-158.34)	87.5 (41.60-184.03)
Visit based continuity	Medium versus low	0.2 (0.14-0.31)	0.2 (0.13-0.31)
	High versus low	0.0 (0.02-0.09)	0.4 (0.03-0.10)
Patient competence	Medium versus low	4.9 (3.18-7.49)	4.7 (3.02-7.50)
	High versus low	10.9 (5.96-20.01)	9.1 ( 5.35-15.63)
Preventive counselling	Medium versus low	3.2 (2.14-4.72)	2.5 (1.70-3.98)
	High versus low	4.7 (3.13-7.02)	3.6 (2.37-5.73)
Physical examination	Medium versus low	4.4 (2.87-6.86)	4.7(3.31-6.80)
	High versus low	7.4 (4.39-12.62)	8.2 (5.25-12.87)
communication	Medium versus low	4.9 (3.28-7.28)	4.8 (3.08-7.47)
	High versus low	11.5(6.88-18.08)	16.4 (9.61-28.04)
Interpersonal treatment	Medium versus low	4.7 (3.27-6.74)	5.0 (3.42-7.41)
	High versus low	12.3 (7.66-19.87)	12.3 (7.38-20.41)
Trust	Medium versus low	3.1 (2.10-4.52)	3.1 (2.07-4.82)
	High versus low	5.4 ( 3.55-8.13)	4.5 (2.84-7.01)

The reference category for service provider is PHFWs; the reference category for perceived quality of care is low quality and is always equal to one.

Models for integration and longitudinal continuity were excluded because of sample size violation.

Other covariates included in each of the models were socio-economic status, previous visit to a CHW or PHFW, education level, type of disease and duration between the interview and the health provider visit.

**Table 7:** Modified Poisson regression models for the association the between the predicted quality score and both service providers and visit outcomes (N= 753).

Predicted perceived quality	unadjusted quality IRR	p-value	adjusted quality IRR	p-value
CHWs versus PHFWs	3.3 (2.73-4.02)	<0.001	3.10 (2.53-3.81)	<0.001*
Child recovered versus not	1.70 (1.37- 2.10)	<0.001	1.56 (1.26-1.93)	<0.001*
Needed more treatment versus not	0.90 (0.66-1.23)	0.512	1.52 (1.01-2.28)	0.043*
Still on treatment versus not	0.72 (0 .50-1.06)	0.089	1.15 (0.75-1.77)	0.513

* Significant p-value

Other covariates included in each of the models were socio-economic status, previous visit to CHW or PHFW, education level, type of disease and duration between the interview and the health provider visit.

## Discussion

Results from this study show higher ratings for PQC received from CHWs compared to care received from PHFWs in all quality domains, except for continuity. The association between quality of health care received and health provider choice has been previously debated [38,39,40]. Campbell argues that quality is best applied to the individual user who accesses the health structure, interacts with health providers and receives holistic care [10]. This same concept has been used to explain the differences in scores observed in this study.

In terms of structure, iCCM is inherently associated with improved financial and geographical access as it uses CHWs who provide free health services [2,27]. Previous studies have shown that accessibility, affordability, and free medicines are all associated with better PQC [39,41]. Although both PHFWs and CHWs offer free services, differences in costs are likely to occur due to some differences in financing mechanisms used. For example a study on user satisfaction with quality of health care in Uganda reported on unexpected health costs incurred by patients at the hospitals in the form of unforeseen investigations [21]. Corruption in form of under the table user-fees is also well documented among PHFWs in Uganda and other low income countries [42,43,44]. Organisational access differs with respect to flexibility in working hours whereby the home based CHWs are more likely to be flexible compared to PHFWs, a characteristic which is valued by caretakers [27]. In Uganda staff absenteeism in health facilities is well documented, leading to facilities being unmanned during opening hours a phenomenon that has been termed as “the quiet corruption” by the World Bank (The World Bank, 2010). 

Longitudinal continuity was rated lower for CHWs than for PHFWs, probably because iCCM was inaugurated in Uganda as late as June 2010, while primary health facility units might have had the same staff for longer durations. Some studies have argued that visit based continuity can improve PQC through the establishment of provider-user relationships [14,20]. It is therefore not clear why CHWs were rated lower than PHFWs despite implied higher continuity. It is possible that CHWs’ inability to treat a child who gets worse on treatment (as they are advised to refer) might be a contributing factor.

CHWs operate within their own community and are therefore more likely to behave as expected by the community members and to have established trusting relationships with caretakers [27,45]. They are more likely to communicate better with their already known subjects during a clinical interaction session leading to higher scores for process measures of quality. Trust is also known to have a role in health seeking behaviour with respect to provider choice [10,20,46].

Satisfaction with health provider as an outcome was rated higher among the PHFWs. It is possible that caretakers are more comfortable with the education level and professional skills of PHFWs. It is also likely that they appreciate getting several opinions from different health workers between illness episodes. However, there is a need to dig deeper into the low satisfaction despite high PQC.

Study limitations and methodological considerations included recall bias which was minimized through use of a two week period between provider visits and interview. Quality ratings vary with the interval between the interview and the visit to the health provider [47]. Data obtained from cluster samples have been criticized for lack of representability compared to individual samples [48], however cluster robust standard errors were used to adjust for clustering at health worker level. The study tool was previously validated in non-African contexts and efforts were made to adapt the questionnaire through comparison with questions from the Uganda service provision survey. The self-selection of individuals receiving care from health facilities and CHWs in the study might have created an unavoidable bias as PHFWs might be seeing sicker children however attempts were made to recruit children with similar diseases. There was an inevitable possibility of caretakers perceiving quality as high for CHWs due to mere friendship. The study was also not able to capture the cadre of staff at the primary health facility level where more often than not several cadres of staff exist.

## Conclusion

In an ‘ideal’ world of universal health coverage (UHC), access to the best clinical and interpersonal quality of care is everyone’s right. In this resource constrained setting, caretakers’ PQC was significantly higher for CHWs compared to PHFWs. This finding suggests that it is viable to improve access to care for sick children through use of CHWs as their services are well perceived. CHWs could thus play an important interim role in achieving UHC by addressing quality gaps in the areas of access to health care and patient- provider interactions where they score highly. Since PHFWs supervise CHWs and manage referred children, strategies that improve the health provider and patient relationship should be of concern in health system strengthening. 
